# Point-of-care ultrasound in emergency department sepsis and septic shock: a pragmatic framework for source-oriented assessment and physiologic reassessment

**DOI:** 10.3389/fmed.2026.1919161

**Published:** 2026-09-22

**Authors:** Weiting Chen, Yangtian Ye, Zi Ye, Xiaoshuang Jiang, Jiuzhou Lin, Min Tang, Yongwei Song

**Affiliations:** 1 Department of Emergency Medicine and Intensive Care Medicine, The First People’s Hospital of Linhai, Taizhou, Zhejiang, China; 2 Department of Clinical Medicine, Hangzhou Medical College, Hangzhou, Zhejiang, China; 3 Department of Rheumatology and Immunology, Hangzhou Medical College, Hangzhou, Zhejiang, China

**Keywords:** emergency department, focused cardiac ultrasound, lung ultrasound, physiologic reassessment, point-of-care ultrasound, sepsis, septic shock

## Abstract

In emergency department (ED) sepsis and septic shock, early management is often dominated by two bedside uncertainties: identifying a clinically plausible infection source and determining whether additional fluid is likely to improve perfusion or worsen pulmonary or systemic congestion. Point-of-care ultrasound (POCUS) may contribute to both questions when it is used as a trigger-based, time-boxed adjunct rather than as a comprehensive screening test or stand-alone diagnostic adjudicator. This article presents a pragmatic narrative review of multiorgan POCUS in adult ED sepsis and septic shock, based on a focused, non-systematic search of major databases and a synthesis of sepsis guidelines, landmark resuscitation trials, and clinically relevant reviews and representative studies addressing source-oriented assessment, shock phenotyping, fluid responsiveness, and congestion assessment. Particular attention was given to separating diagnostic capability and physiologic plausibility from demonstrated clinical utility and patient-centered outcome benefit. Priority was given to adult ED literature; intensive care unit (ICU), perioperative, and physiologic studies were used only when ED-specific evidence was limited and the underlying principles were clinically transferable. The proposed framework is organized around two linked arms. First, a source-oriented scan may support pneumonia or pleural complication as a working source and may identify biliary disease, obstructive urinary infection, urinary retention, or drainable soft-tissue infection when clinical features make these sources plausible. Its intended role is to trigger formal imaging, drainage planning, specialist consultation, or source-control pathways, not to prove the source at the bedside. Second, physiologic reassessment can use focused cardiac ultrasound (FoCUS), dynamic flow-based assessment such as passive leg raise with left ventricular outflow tract velocity-time integral (PLR-LVOT VTI), and serial lung ultrasound to provide context for fluid and vasopressor decisions after the initial resuscitation step. Venous Doppler and the venous excess ultrasound (VExUS) framework are discussed as optional advanced congestion assessments, not as routine ED requirements, because ED sepsis-specific evidence remains limited. Overall, current evidence supports POCUS mainly as an adjunct for bedside clarification, escalation support, and physiologic reassessment; direct evidence that POCUS-guided ED sepsis pathways improve patient-centered outcomes remains limited. POCUS should therefore be used as a decision-support tool, not a stand-alone adjudicator or a reason to delay antimicrobials, CT, formal imaging, or specialist involvement.

## 1 Introduction

Sepsis and septic shock remain among the most demanding emergencies encountered in acute care. Despite major advances in antimicrobial therapy, hemodynamic support, and critical care delivery, they continue to cause substantial short-term mortality and long-term morbidity (1–4). Sepsis-3 reframed sepsis as life-threatening organ dysfunction caused by a dysregulated host response to infection (1), and contemporary international guidance has moved practice away from rigid, uniform resuscitation toward earlier recognition, timely antimicrobial therapy, source control, and individualized hemodynamic management (2). In the ED, however, the opening phase of care is still dominated by two recurring bedside uncertainties: the source of infection is not always immediately apparent, and the physiology of shock is often more complex than isolated vasodilation. In many patients, fluid administration may be both necessary and potentially harmful, particularly when myocardial dysfunction, right-sided loading abnormalities, or venous congestion coexist (2, 3, 5).

POCUS fits naturally into this setting because it can be performed immediately, repeated as the clinical picture evolves, and interpreted alongside physical examination, laboratory data, and response to treatment (6–9). Recent reviews focused on sepsis have described multiorgan approaches that integrate lung and pleural ultrasound, focused cardiac views, abdominal and renal imaging, and selected Doppler techniques to address both source-oriented assessment and hemodynamic decision-making (9–12). However, diagnostic visualization and physiologic plausibility should not be conflated with proven clinical utility. Evidence that POCUS-guided ED sepsis pathways improve patient-centered outcomes remains limited. A negative or equivocal examination should therefore guide escalation when needed rather than provide false reassurance, and a positive finding should generally be treated as a trigger for confirmation, consultation, or source-control planning rather than as definitive proof of the diagnosis.

Although the growing literature on multiorgan POCUS in sepsis has broadened understanding of its diagnostic and physiologic applications, much of that literature remains organized either by organ system or by ultrasound modality (12). Less attention has been paid to how POCUS can be structured around the practical bedside decisions that dominate the first hours of ED sepsis care: when to scan, which patients are most likely to benefit, what finding should trigger action, and when uncertainty should lead directly to CT, formal echocardiography, radiology-performed ultrasound, or specialist consultation.

Accordingly, this review addresses two bedside questions that are central to early ED management of adult sepsis and septic shock. First, in which clinical situations can multiorgan POCUS help identify a plausible and actionable infectious source? Second, after initial resuscitation has begun, how can ultrasound findings support physiologic reassessment without overstating the current evidence for outcome benefit? To answer these questions, the review is organized around two linked arms: source-oriented triage and physiologic reassessment. The overall framework is summarized in Figure 1.

**Figure 1:**
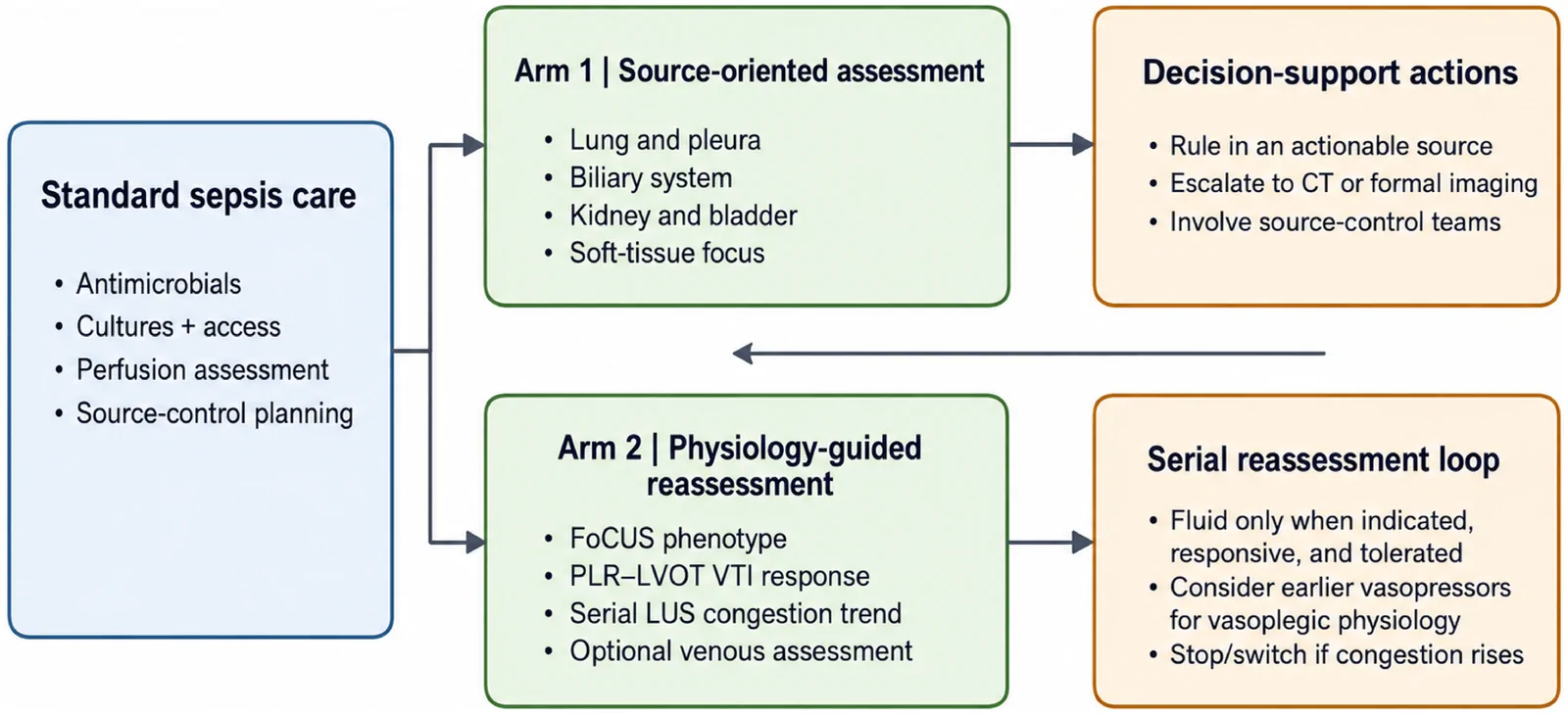
Two-arm ED POCUS framework for source-oriented assessment and physiologic reassessment in sepsis and septic shock. POCUS runs in parallel with standard sepsis care and supports selected escalation and reassessment decisions without replacing antimicrobials, source control, comprehensive imaging, or clinical judgment. CT, computed tomography; ED, emergency department; FoCUS, focused cardiac ultrasound; LUS, lung ultrasound; LVOT, left ventricular outflow tract; PLR, passive leg raise; POCUS, point-of-care ultrasound; VTI, velocity-time integral.

## 2 Methods

This review was designed as a pragmatic narrative evidence synthesis to inform bedside decision-making in adult ED sepsis and septic shock. A focused, non-systematic search of the published literature was undertaken in PubMed, Embase, Scopus, and Web of Science from database inception to 6 May 2026, after publication of the 2026 Surviving Sepsis Campaign (SSC) guideline, to identify major sepsis definitions and guidelines (1, 2), landmark and contemporary trials relevant to fluid and vasopressor strategy (5, 13, 14), and key systematic reviews, focused reviews, and representative original studies addressing multiorgan POCUS, source-oriented assessment, shock phenotyping, fluid responsiveness, congestion assessment, and source-control escalation (9–12). The reproducible core database search (Search A) combined three mandatory concept blocks: sepsis/septic shock, ultrasound/POCUS, and source-oriented terms covering source control, pulmonary or pleural infection, biliary infection, urinary obstruction, abscess, and soft-tissue infection. The complete database-specific Search A strategies are provided in Supplementary Table S1. The four operational Search A strategies were run on 6 May 2026 without language restrictions, yielding 175 records in PubMed, 3,305 in Embase, 3,106 in Scopus, and 469 in Web of Science. In addition to Search A, non-prespecified, iterative supplementary searches were used across the same databases to identify landmark and clinically relevant hemodynamic literature on FoCUS, fluid responsiveness, congestion, and resuscitation. These exploratory searches combined sepsis or septic shock terms with topic-specific combinations including focused cardiac ultrasound or echocardiography; passive leg raising, LVOT VTI, or fluid responsiveness; lung ultrasound, B-lines, or fluid tolerance; and venous Doppler, VExUS, or venous congestion. Search wording was refined iteratively in response to the literature retrieved, and backward and forward citation chaining was used to identify landmark and representative studies. Because these supplementary searches were not protocolized or intended to provide exhaustive coverage, complete search histories and aggregate yields were not prespecified or retained; they are therefore reported transparently as a limitation rather than presented as fully reproducible systematic searches. During synthesis, evidence was explicitly interpreted according to whether it supported diagnostic accuracy, physiologic rationale, workflow escalation, or patient-centered outcome benefit.

Priority was given to adult ED literature and to studies with direct bedside implications. When ED-specific evidence was limited, ICU and perioperative studies were considered only if the underlying physiologic principles were judged reasonably transferable to ED practice. The synthesis deliberately distinguishes between ED-specific evidence and broader physiologic or critical care evidence used as contextual support. This distinction is particularly important in the resuscitation section, where FoCUS and dynamic flow-based assessment have stronger physiologic support than direct evidence from ED sepsis treatment trials, and where venous Doppler/VExUS evidence remains largely observational and non-ED-specific. Therefore, the hemodynamic framework proposed here should be interpreted as a set of practice considerations for selected bedside questions rather than as a validated ED sepsis treatment algorithm.

A narrative rather than systematic review approach was chosen because the aim was not exhaustive evidence quantification, formal comparative-effectiveness assessment, or guideline development, but a practice-oriented synthesis of heterogeneous evidence across organ systems and ultrasound applications around a limited number of high-impact bedside questions. Formal systematic-review procedures, risk-of-bias assessment, and meta-analytic methods were not applied. To improve bibliographic precision in this revision, high-yield diagnostic and hemodynamic references were rechecked against PubMed and journal records. Because this work synthesized published literature only and did not involve human participants, animal experiments, or identifiable patient data, ethics committee approval was not required. Generative AI was not used as an evidence source or to make final study-selection decisions; its role in manuscript preparation is disclosed in the Generative AI statement.

Study selection was guided by the prespecified bedside questions and clinical relevance rather than by exhaustive systematic-review procedures. Records were considered when they addressed adult ED sepsis or septic shock, source-oriented POCUS, shock phenotyping, fluid responsiveness, congestion assessment, or clinically transferable acute-care physiology. Studies were not retained for detailed synthesis when they did not inform one of these bedside questions or when the population, setting, or technique was not reasonably transferable to adult ED practice. When multiple publications addressed the same question, priority was given to current guidelines, systematic reviews, landmark randomized trials, and representative recent original studies. The literature search involved Yangtian Ye and Zi Ye, and literature screening/data curation involved Xiaoshuang Jiang, consistent with the author-contribution statement; no claim of independent duplicate systematic screening is made.

## 3 Pragmatic ED framework at a glance

Before each ultrasound domain is discussed in detail, this review defines a minimum ED-facing approach. POCUS should run in parallel with standard sepsis care. Cultures, antimicrobials, vascular access, lactate or perfusion assessment, and early source-control planning should not wait for ultrasound. The 2026 SSC guideline frames sepsis and septic shock as medical emergencies; therefore, bedside ultrasound should not delay immediate antimicrobials in septic shock or probable/definite sepsis, time-limited rapid investigation when sepsis is possible without shock, early fluid resuscitation, vasopressor initiation, or source-control escalation (2). The examination should also be selected by a clinical trigger rather than performed as a complete multiorgan search in every patient with possible sepsis. The purpose is to clarify a specific bedside uncertainty, not to provide a universal screening pathway. The suggested workflow is intended for emergency physicians and acute-care clinicians with established competency in core POCUS; advanced Doppler assessments require additional training, local quality assurance, and escalation pathways.

For source localization, lung ultrasound is most useful when respiratory infection, hypoxemia, unexplained dyspnea, abnormal chest examination, or pleural complication is clinically plausible. Biliary POCUS is most useful in patients with right upper quadrant pain, jaundice, cholestatic laboratory abnormalities, or suspected cholangitis. Renal and bladder POCUS is most useful when urinary sepsis is accompanied by flank pain, acute kidney injury, oliguria, urinary retention, stone history, or persistent instability. Soft-tissue ultrasound is most useful when focal pain, swelling, erythema, fluctuance, or concern for deep infection makes a drainable collection or necrotizing process clinically relevant. In each domain, a positive scan should trigger action, whereas a negative or equivocal scan should not exclude serious infection when clinical concern persists.

For hemodynamic assessment, FoCUS, lung ultrasound, and dynamic flow-based testing are most relevant when hypotension or hypoperfusion persists after the initial resuscitation step, when the response to fluid is unclear, or when the patient has features that increase concern for fluid intolerance, such as known heart failure, severe hypoxemia, right ventricular loading risk, renal failure, or rising oxygen requirement. Reassessment should be linked to an intervention: after a cautious fluid bolus, passive leg raise, vasopressor initiation or escalation, ventilatory change, or clinical deterioration. A repeat check after approximately 5–15 min can determine whether stroke-volume surrogates, pulmonary findings, and congestion markers are moving in the expected direction. This suggested workflow is intended to support bedside decisions and escalation; it is not a proven outcome-improving ED sepsis pathway.

## 4 Source-oriented infection-focus assessment in the ED

### 4.1 Why source-oriented triage is an adjunctive contribution of POCUS in early sepsis care

In early sepsis management, timely antimicrobial therapy is essential, but antibiotics alone may be insufficient when a source-control problem is present (2). The 2026 SSC guideline emphasizes rapid evaluation for anatomical diagnoses or sources of infection requiring emergent source control and suggests early source control, ideally within 6 h of diagnosing sepsis or septic shock when a controllable source is present (2). In this context, the value of POCUS is not that it replaces CT, formal echocardiography, radiology-performed ultrasound, or specialist assessment. It also should not be presented as proven to improve patient-centered outcomes or as the main reason that broad-spectrum antimicrobials are given earlier, because empiric antibiotics usually should begin before the source is fully confirmed. Its more defensible ED contribution is source-control triage: identifying selected findings that may move the team toward formal imaging, drainage, urologic or biliary decompression, procedural intervention, or urgent consultation when the clinical picture already makes that source plausible (9–12).

In this review, the term source-oriented refers to a brief, time-boxed early ultrasound strategy that prioritizes infectious foci most likely to alter immediate diagnostic escalation or source-control planning. It does not mean that every septic patient should undergo a complete lung, biliary, renal, bladder, and soft-tissue examination. Such a nonselective approach risks becoming a low-yield fishing expedition. The more practical approach is trigger-based: choose the module that matches the patient's symptoms, examination, laboratory pattern, and pretest probability, then stop or escalate once the bedside question has been answered. If the examination is negative or indeterminate but the patient remains unstable, the appropriate next step is usually escalation to definitive imaging and/or specialty consultation rather than repeated low-yield bedside scanning.

### 4.2 Lung and pleura: informative but not fully specific in ED sepsis

Lower respiratory tract infection is among the most common causes of sepsis and septic shock, and LUS is often the fastest bedside tool for determining whether the chest is a plausible source (8, 12, 15, 16). Its clinical effect, however, depends on context. In an obviously hypoxemic patient with clinically apparent pneumonia, LUS may not change the first antibiotic dose or the immediate need for oxygen or ventilatory support. Its stronger role is in patients with an uncertain respiratory source, discordant examination and chest radiography, suspected pleural complication, unexplained hypoxemia, or concern that pulmonary edema or ARDS-like physiology is being mistaken for infection. Meta-analytic evidence suggests that LUS performs well in adult pneumonia, particularly when chest radiography is delayed, nondiagnostic, or discordant with the clinical picture (15).

To remain useful in septic patients, LUS should be brief and structured, for example across 8–12 zones. In most cases, the examination should focus on patterns with immediate management relevance. Focal subpleural consolidation with dynamic air bronchograms supports pneumonia, and dynamic air bronchograms can help distinguish infectious consolidation from resorptive atelectasis in selected settings (17, 18). Yet this sign should not be interpreted in isolation. Atelectasis, aspiration-related consolidation, ARDS-related dependent consolidation, pulmonary infarction, and noninfectious inflammatory lung disease may overlap with infectious patterns, particularly when the lung is diffusely abnormal or the patient is receiving mechanical ventilation (18, 19). Similarly, B-lines and pleural line abnormalities may be seen in pulmonary edema, ARDS, interstitial pneumonia, or chronic interstitial lung disease, and therefore they are not specific for bacterial pneumonia (19, 20).

These limitations do not make LUS less useful; they make its role more precise. In ED sepsis, LUS is most actionable when it supports pneumonia as a clinically concordant working source, identifies a complex pleural effusion that may require drainage planning, or reveals competing physiology such as pulmonary edema or pneumothorax (6–8, 12). In these situations, LUS can influence escalation and supportive care: complicated pleural disease may prompt CT or drainage planning, diffuse congestion may prompt reconsideration of further fluid, and a noninfectious pattern may broaden the diagnostic work-up. By contrast, a negative or nonspecific LUS examination should not be used to delay antibiotics, chest imaging, or further evaluation when respiratory infection remains clinically likely (2, 12).

### 4.3 Abdomen and biliary infection: early triage for source-control pathways

Abdominal infection is a frequent cause of severe sepsis, and biliary disease is one of the clearest examples of a clinical scenario in which bedside identification can accelerate a source-control pathway. In a nationwide sepsis source analysis, biliary tract infection represented a smaller proportion of all sepsis sources than respiratory or genitourinary infection, but it still carried clinically meaningful mortality and requires prompt recognition when suspected (16). In the ED, POCUS can rapidly identify gallbladder distension, gallstones with posterior acoustic shadowing, wall thickening, pericholecystic fluid, and a sonographic Murphy sign. When interpreted together, these findings can substantially increase suspicion for acute cholecystitis and support concern for a biliary source requiring urgent escalation. Current evidence suggests that emergency physician-performed POCUS has high specificity but only moderate sensitivity for acute cholecystitis, which makes it more useful for ruling in disease and expediting management than for excluding it (21).

From a practical ED perspective, focused biliary POCUS should be reserved for septic patients with a reasonable biliary pretest probability, such as right upper quadrant pain, jaundice, cholestatic liver tests, recent biliary intervention, or suspected cholangitis. If the scan supports a biliary source, particularly in the setting of suspected obstruction or complicated cholecystitis, its role is not to replace definitive imaging or specialist assessment, but to move them forward. In that setting, the most appropriate next step is earlier escalation to formal imaging and urgent gastroenterology or surgical consultation, consistent with the principle that bedside ultrasound should accelerate source control rather than stand in for it (2, 21).

### 4.4 Urinary tract source: keeping obstruction in the foreground

Urinary tract infection is a common source of sepsis. In the ED, the key ultrasound question is usually not simply whether infection is present, but whether obstruction, retention, or another anatomic problem coexists and urgent decompression may be required. Genitourinary infection is a major sepsis source in population-level data (16). In obstructive acute pyelonephritis with upper urinary tract calculi requiring emergency drainage, one cohort found objective evidence of sepsis in 63.4% of events and septic shock in 20.8% (22). These data support a focused renal and bladder scan in selected patients with suspected urinary sepsis and flank pain, acute kidney injury, oliguria, urinary retention, stone history, recurrent UTI, or persistent instability. In such patients, hydronephrosis or urinary retention should immediately raise concern for a source-control problem and prompt earlier urologic consultation, definitive imaging, and decompression planning rather than allowing treatment to stop at antibiotics alone.

From an ED standpoint, renal and bladder POCUS is therefore best understood as a source-control triage test rather than a stand-alone diagnostic endpoint. A positive examination, particularly hydronephrosis in an unstable patient or bladder retention in a patient with urinary sepsis, should prompt urgent escalation. By contrast, a negative scan does not exclude complicated infection, pyelonephritis, infected stone, renal abscess, or another intra-abdominal source, and CT may still be required when hemodynamic instability, organ dysfunction, or persistent diagnostic uncertainty remains (2, 22, 23).

### 4.5 Skin and soft tissue infection, including necrotizing infection

Skin and soft tissue infection is common in the ED, but physical examination alone does not always reliably distinguish cellulitis from abscess. In this setting, soft-tissue POCUS is most useful when the management question is focused: is there a drainable collection at the symptomatic site? This includes patients with focal swelling, pain, erythema, induration, equivocal fluctuance, prior failed therapy, or high-risk anatomy where bedside drainage decisions are uncertain. Systematic review and meta-analysis data in ED populations have shown good diagnostic accuracy for differentiating abscess from cellulitis (24). Its value, therefore, lies not only in refining the diagnosis but also in moving management more quickly toward either procedural source control or continued medical treatment.

Necrotizing infection presents a different and more consequential problem. Although it is relatively uncommon, the consequences of delayed recognition are substantial. Ultrasound findings such as fascial thickening, perifascial fluid, and subcutaneous gas may increase suspicion, and recent systematic review data suggest diagnostic potential when these findings are interpreted together with the broader clinical picture (25). However, the practical role of ultrasound in this setting is fundamentally different from its role in uncomplicated soft-tissue infection. Bedside findings may support earlier recognition and escalation, but they cannot safely rule in or rule out necrotizing infection in a high-risk patient. If pain is disproportionate, toxicity is present, crepitus is suspected, soft-tissue gas is seen, or clinical concern remains substantial, ultrasound should occur only if it does not delay CT, broad antimicrobial therapy, or immediate surgical consultation.

### 4.6 A practical source-first ED approach in the first 10 min

In early ED sepsis care, POCUS is most useful when it runs in parallel with standard resuscitative care rather than after it. While antimicrobials, cultures, vascular access, and other bundle elements are being initiated, the operator can perform a brief, time-boxed examination directed by clinical triggers. The lung and pleura should be scanned when respiratory source or pleural complication is plausible. The right upper quadrant should be scanned when biliary symptoms or cholestatic markers are present. The kidneys and bladder should be scanned when urinary source is suspected and obstruction or retention would change management. The soft-tissue site should be scanned when the bedside question is whether a drainable collection or high-risk deep infection is present (2, 8–10).

In practical terms, a supported source should prompt action only when it is clinically concordant, an uncertain result should prompt escalation to CT or radiology-based imaging, and an alternative diagnosis should shift priorities accordingly. Framed in this way, the source-oriented examination serves as an early triage tool for definitive imaging, specialist involvement, and source-control planning rather than as a stand-alone diagnostic endpoint. Key features of the source-oriented framework are summarized in Table 1.

**Table 1:** Source-oriented ED POCUS framework: scan targets, action-supporting findings, immediate implications, and escalation triggers.

**Domain**	**Clinical trigger and focused scan target(s)**	**Action-supporting findings (examples)**	**Immediate ED implication**	**If negative/indeterminate but concern persists**
Lung and pleura	Suspected respiratory source, hypoxemia/dyspnea, abnormal chest examination, discordant CXR, or pleural complication; 8-12-zone LUS	Focal consolidation with dynamic air bronchograms; complex pleural effusion; diffuse B-lines or pleural abnormalities suggesting edema/ARDS-like or interstitial pattern	Treat respiratory source as a working diagnosis only when clinically concordant; reassess oxygenation/ventilation; consider pleural drainage planning or CT escalation when complicated effusion is suspected	Chest radiography and/or CT when respiratory source remains likely, shock persists, or pleural complication cannot be excluded
Right upper quadrant/biliary system	Right upper quadrant pain, jaundice, cholestatic tests, recent biliary intervention, or suspected cholangitis; gallbladder +/- common bile duct when feasible	Gallstones with shadowing, wall thickening, pericholecystic fluid, sonographic Murphy sign	Suspect biliary source; expedite formal biliary imaging and GI/surgical consultation	Formal biliary imaging and urgent specialist input when cholangitis, obstruction, or complicated cholecystitis remains a concern
Kidney/urinary tract	Suspected urinary source with flank pain, AKI, oliguria, urinary retention, stone history, recurrent UTI, or persistent instability; kidneys and bladder	Hydronephrosis, obstruction pattern, urinary retention	Suspect obstructive urinary source; expedite urologic consultation, CT, and decompression planning	CT and further urologic evaluation when instability, organ dysfunction, or concern for complicated infection persists
Skin and soft tissue	Focal pain, swelling, erythema, equivocal fluctuance, failed therapy, or concern for deep infection; area of concern	Drainable abscess; fascial fluid tracking; subcutaneous gas	Proceed toward drainage when appropriate; urgent escalation if necrotizing infection is suspected	Surgical consultation +/- CT when necrotizing infection remains a concern; do not let ultrasound delay operative evaluation in high-risk patients

AKI, acute kidney injury; ARDS, acute respiratory distress syndrome; CT, computed tomography; CXR, chest radiography; ED, emergency department; GI, gastroenterology; LUS, lung ultrasound; POCUS, point-of-care ultrasound; UTI, urinary tract infection*.*

## 5 ED hemodynamics and fluid strategy: a physiology-guided reassessment approach

### 5.1 Why ultrasound may refine fluid versus vasopressor decisions in selected patients

Early sepsis trials were built around aggressive fluid loading and protocolized resuscitation targets. However, subsequent multicenter randomized trials showed that rigid early goal-directed therapy did not outperform contemporary usual care once timely antimicrobial therapy and earlier recognition had become standard practice (5, 13, 26, 27). The 2026 SSC guideline still treats sepsis-induced hypoperfusion and septic shock as medical emergencies and suggests at least 30 mL/kg of intravenous crystalloid within the first 3 h, while also noting that individual patient characteristics and context should inform the initial volume (2). After that initial resuscitation step, the guideline allows either a liberal or restrictive approach in patients with persistent hypoperfusion based on individual patient and health-system factors and suggests using dynamic measures over physical examination or static measures alone for ongoing fluid guidance (2). More recent trials have examined fluid volume and vasopressor timing more directly. CLASSIC showed that a restrictive post-initial fluid strategy did not worsen 90-day mortality in ICU septic shock, whereas CLOVERS found no overall mortality advantage for either a restrictive or liberal strategy in early sepsis-induced hypotension after an initial fluid step (14, 28). These findings should not be read as proof that IV fluid is generally harmful. They more appropriately support clinical equipoise after initial resuscitation and reinforce the need for reassessment before repeated automatic fluid boluses. This question is especially relevant in the ED, where bedside classification of undifferentiated hypotension remains imperfect even before ultrasound is introduced (29). At the same time, the direct ED evidence base for physiology-guided hemodynamic decision-making remains narrower than the evidence supporting ultrasound for source clarification, which means that part of the discussion that follows necessarily draws on broader physiologic and critical care literature.

What ultrasound adds in this setting is not certainty or a proven outcome-improving algorithm, but better physiologic context. In this review, physiology-guided refers to the use of ultrasound findings to refine bedside reassessment regarding fluid, vasopressor, or inotropic support based on the patient's current hemodynamic profile rather than on hypotension alone. Its clinical value depends less on any single image than on the integration of FoCUS, LUS, and dynamic Doppler-based testing into a repeatable reassessment loop (9–12). Framed in this way, ultrasound may help move fluid and vasopressor decisions away from reflexive protocolization and closer to individualized bedside phenotyping, while still requiring careful attention to the distinction between ED-based evidence and extrapolations from non-ED populations.

### 5.2 FoCUS shock phenotyping in sepsis: a minimum dataset that may inform management

In the ED, a feasible FoCUS examination should answer a limited number of practical questions rather than attempt a comprehensive echocardiographic assessment or screen every unstable patient for every possible catastrophic diagnosis. It is most useful when shock is persistent or unexplained, the response to initial fluid is poor, myocardial dysfunction is suspected, right ventricular strain is clinically plausible, hypoxemia is disproportionate, or additional fluid could plausibly worsen congestion. At minimum, FoCUS should screen for left ventricular systolic depression, whether related to septic cardiomyopathy or clinically relevant preexisting dysfunction. It should also assess the right ventricle for dilation or strain, which may raise concern for pulmonary embolism, pulmonary hypertension, or severe ARDS-related loading conditions. In addition, it should exclude pericardial effusion with tamponade physiology and provide a cautious qualitative impression of preload and intravascular filling status, interpreted in context rather than inferred from inferior vena cava findings alone.

FoCUS phenotypes should not be interpreted as fixed diagnoses. A hyperdynamic LV may accompany distributive vasodilation but may also coexist with hypovolemia; depressed LV systolic function may represent acute septic cardiomyopathy or chronic cardiomyopathy; and RV dilation or strain may reflect acute pulmonary embolism, chronic pulmonary hypertension, ARDS-related loading, or mechanical ventilation. Apparent chamber size and function are also influenced by preload, afterload, ventilator pressures, rhythm, image quality, and operator technique. FoCUS findings should therefore be integrated with clinical history, serial response, and formal echocardiography when management depends on a precise diagnosis.

This focused dataset is clinically important because septic shock is often vasoplegic, but myocardial dysfunction and right ventricular failure are not uncommon and may evolve during the first several hours of illness (3, 10). In these settings, repeated fluid boluses may worsen pulmonary edema or venous congestion without meaningfully improving perfusion, which can make earlier vasopressor support and, in selected cases, inotropic evaluation more consistent with the observed physiologic profile (14, 28, 30, 31). The 2026 SSC guideline also supports peripheral vasopressor initiation rather than delaying vasopressors until central venous access is secured, recommends norepinephrine as the first-line vasopressor, and suggests inotropes when septic shock is accompanied by cardiac dysfunction and persistent hypoperfusion despite adequate volume status and arterial pressure (2). These recommendations do not validate an ultrasound-guided treatment algorithm, but they make timely escalation feasible when POCUS suggests poor fluid tolerance or cardiac dysfunction. Randomized ED data in undifferentiated hypotension suggest that protocolized POCUS performs reasonably well as a diagnostic rule-in tool, but it has not demonstrated clear superiority over standard assessment and has not improved short-term resuscitation markers (32, 33). Because these ED trials were not sepsis-specific treatment studies, they are best interpreted as supporting the bedside phenotyping value of FoCUS rather than as direct evidence that ultrasound-guided hemodynamic management improves outcomes in ED sepsis. Common FoCUS-based phenotypes and potential ED reassessment pivots are summarized in Table 2.

**Table 2:** FoCUS phenotypes in sepsis: bedside patterns and potential ED reassessment pivots.

Phenotype pattern	Suggestive ultrasound features	Clinical implication	Typical ED management pivot
Vasodilatory-dominant after the initial fluid step	Hyperdynamic or preserved LV function, no major RV strain, minimal congestion	Persistent hypotension may be more vasoplegic than preload-responsive	Supports consideration of earlier norepinephrine over further empiric fluid when dynamic testing and congestion assessment remain unfavorable
LV systolic dysfunction/septic cardiomyopathy	Depressed LV systolic function, often with pulmonary congestion on LUS	Higher risk of fluid intolerance; perfusion may not improve with repeated boluses	Supports consideration of vasopressor support and selected inotropic evaluation; avoid assuming further empiric fluid will improve perfusion
RV strain or RV loading	RV dilation and/or septal flattening, often with severe hypoxemia	Consider pulmonary embolism, pulmonary vascular load, or ARDS-related RV dysfunction	Supports broader evaluation of myocardial dysfunction and congestion rather than assuming further fluid is beneficial by default
Obstructive or coexisting nonvasoplegic process	Pericardial effusion/tamponade physiology or marked obstructive RV pattern	Potential alternative or coexisting obstructive process	Supports urgent cause-directed escalation over a default sepsis-only framing

ARDS, acute respiratory distress syndrome; ED, emergency department; FoCUS, focused cardiac ultrasound; LUS, lung ultrasound; LV, left ventricle/left ventricular; RV, right ventricle/right ventricular.

### 5.3 The key principle: distinguishing fluid responsiveness from fluid tolerance

A useful way to frame bedside resuscitation is to separate two related but non-identical questions: whether stroke volume is likely to increase if fluid is administered, and whether the patient can tolerate that fluid without developing clinically harmful congestion. The first question concerns fluid responsiveness; the second concerns fluid tolerance. This distinction recurs across physiologic discussions of sepsis resuscitation and in more focused POCUS-based literature (9, 31, 34), and it is particularly important in the ED, where uncertainty often arises not from a lack of hypotension, but from uncertainty about what additional fluid is likely to achieve. Fluid responsiveness is not, by itself, an indication for fluid administration; any potential increase in stroke volume must be weighed against ongoing perfusion need, fluid tolerance, and the risk of pulmonary or systemic congestion, particularly after the initial resuscitation step.

When the aim is to estimate responsiveness, dynamic tests are generally preferred to static inferior vena cava measurements, a position consistent with the 2026 SSC recommendation to use dynamic measures over physical examination or static measures alone for fluid guidance (2, 31, 34, 35). Among these, PLR is especially attractive because it functions as a reversible autotransfusion and can be interpreted in a practical, bedside manner (35). In the ED, one of the most actionable implementations is PLR-VTI assessment using LVOT VTI, or a comparable aortic Doppler surrogate when available. A meaningful rise in VTI, often in the range of 10%–15% depending on technique, supports the likelihood of responsiveness and may justify a cautious fluid bolus in the appropriate clinical context (34, 35). Recent randomized-trial synthesis suggests that dynamic fluid-responsiveness-guided resuscitation may improve some outcomes in sepsis and septic shock, but effects across ICU mortality, length of stay, organ support, and specific measurement modalities remain uncertain (36). Therefore, PLR-VTI should be framed as a decision-support tool, not as a mandatory ED sepsis requirement. By contrast, static inferior vena cava findings may still provide supportive context, but they should not be used in isolation either to mandate further fluid administration or to deny it.

Assessment of fluid tolerance should proceed in parallel rather than after responsiveness has already been judged. LUS can identify increasing B-lines or a rising interstitial burden, but these findings remain nonspecific and must be interpreted with baseline lung disease, ARDS, pneumonia, and ventilator effects in mind (19, 20). Venous Doppler and the VExUS framework may provide a more structured signal that systemic congestion is developing (37, 38). Accordingly, this review frames VExUS conservatively for ED sepsis. In the ED, venous Doppler and VExUS should not be interpreted as routine requirements for sepsis resuscitation. They may be considered as optional advanced congestion assessments in selected patients when operator expertise, time, and image quality are sufficient. Current ED sepsis-specific evidence remains limited, and recent septic shock work remains observational rather than treatment-validating (39). Therefore, these tools should support, rather than determine, fluid-tolerance decisions. A practical summary of physiology-guided responsiveness and congestion tools is provided in Table 3. Interobserver agreement may vary, and technically adequate hepatic and portal venous Doppler acquisition can be difficult in the time-constrained ED environment.

**Table 3:** Adjunctive physiology-guided tools for fluid responsiveness and congestion assessment in ED sepsis.

Tool	Primary bedside question	Main strength	Key ED limitation
Passive leg raise with LVOT VTI or comparable aortic Doppler surrogate	Is the patient likely to increase stroke volume with additional fluid?	Dynamic, reversible, and directly linked to change in flow	Requires Doppler skill and interpretable windows; rhythm/respiratory factors may confound interpretation
Inferior vena cava size and respiratory variation	Provides preload-related context; should not be used alone to decide for or against further fluid	Fast and widely available	Strongly affected by ventilation, RV dysfunction, intra-abdominal pressure, and loading conditions
Lung ultrasound B-line trend	Is pulmonary congestion or interstitial burden increasing?	Rapid, repeatable, and easy to trend serially	Not specific for fluid alone; ARDS, pneumonia, and interstitial lung disease may produce overlapping patterns
Venous Doppler/VExUS	Is systemic venous congestion developing?	Provides a structured congestion signal beyond the lungs	Optional advanced add-on only; ED sepsis evidence remains limited; acquisition and interpretation require additional training

ARDS, acute respiratory distress syndrome; ED, emergency department; LVOT, left ventricular outflow tract; RV, right ventricle/right ventricular; VExUS, venous excess ultrasound; VTI, velocity-time integral.

### 5.4 Evidence-informed fluid strategy: what POCUS may support, but not replace

Guideline and trial evidence suggest that fluid strategy after the initial resuscitation step should be individualized rather than rigidly protocolized. The 2026 SSC guideline continues to support early crystalloid resuscitation for sepsis-induced hypoperfusion or septic shock, but it also explicitly allows either liberal or restrictive strategies after 30 mL/kg when hypoperfusion persists and recommends dynamic measures rather than static assessment alone for ongoing fluid guidance (2). In septic shock managed in the ICU, the CLASSIC trial showed that a restrictive post-resuscitation fluid strategy did not worsen 90-day mortality (14). In patients with early sepsis-induced hypotension, the CLOVERS trial did not demonstrate an overall mortality advantage for either a restrictive or a more liberal strategy, but it reinforced the practical feasibility of a vasopressor-forward approach once the initial resuscitation step had already been taken (28). Ultrasound does not replace the evidentiary role of these guidelines or trials. Its role is narrower and more bedside-oriented: it helps determine which physiologic profile, at a given moment, appears more likely to benefit from cautious additional fluid, earlier vasopressor support, or closer reassessment without immediate further intervention. Guideline and trial-level evidence relevant to this framing is summarized in Table 4.

**Table 4:** Guideline and trial-level evidence informing the resuscitation context for ultrasound integration in sepsis.

Evidence stream/study context	Main message	Implication for POCUS framing
2026 SSC guideline (2)	Sepsis-induced hypoperfusion and septic shock remain emergencies; at least 30 mL/kg crystalloid in the first 3 h is suggested with individual context, followed by individualized liberal or restrictive strategies and dynamic measures when hypoperfusion persists	POCUS should not delay initial guideline-based care; its main role is to support dynamic reassessment, fluid-tolerance assessment, and escalation decisions after or during the initial resuscitation step
EGDT-era randomized trials (5, 13, 26, 27)	Rigid protocolized EGDT did not outperform contemporary usual care	Supports individualized physiology-based assessment rather than fixed resuscitation targets
CLASSIC trial (14)	A restrictive post-resuscitation fluid strategy did not worsen 90-day mortality in ICU septic shock	Supports caution with automatic ongoing fluid loading after the initial resuscitation step
CLOVERS trial (28)	After an initial fluid step, restrictive and more liberal strategies showed no overall mortality difference in early sepsis-induced hypotension	POCUS may help identify patients who appear less likely to benefit from further fluid and more compatible with earlier vasopressors
Early norepinephrine trial (46)	Phase II data support earlier vasopressor use in selected septic shock patients	Supports consideration of earlier vasopressor use when ultrasound does not support further fluid benefit
Heart failure and other high-risk observational data (40–45)	Evidence is heterogeneous; some studies do not show clear harm from guideline-concordant early fluids, whereas others associate larger volumes with respiratory support in high-risk cohorts	Avoid reflexive under-resuscitation based only on comorbidity; use POCUS to reassess current physiology and tolerance rather than to justify automatic fluid restriction
ED POCUS shock trials/diagnostic analyses (32, 33)	Protocolized POCUS improves bedside phenotyping and rule-in diagnosis more than short-term outcome metrics	Supports early hemodynamic characterization, not a guaranteed outcome-improving intervention
Note	These guidelines and trials inform the physiologic and resuscitation context in which ultrasound may be integrated, rather than directly validating ultrasound-guided sepsis protocols.	

CLASSIC, conservative versus liberal approach to fluid therapy of septic shock in intensive care; CLOVERS, crystalloid liberal or vasopressors early resuscitation in sepsis; ED, emergency department; EGDT, early goal-directed therapy; ICU, intensive care unit; POCUS, point-of-care ultrasound; SSC, surviving sepsis campaign.

Evidence in patients traditionally considered volume sensitive also argues against a reflexively low-fluid strategy. Observational studies in patients with heart failure or reduced left ventricular ejection fraction report mixed findings, including data associating early bundle-compliant fluids with improved survival, data showing no clear increase in adverse outcomes when patients with reduced ejection fraction received similar early fluid volumes, and data linking higher volumes with intubation in heterogeneous high-risk ICU cohorts (40–45). These studies are not definitive and should not be treated as a mandate for liberal fluids. They do support a practical caution: POCUS should help identify current physiology and fluid tolerance, but it should not be used as a blanket reason to deny initial resuscitation in patients with heart failure, kidney disease, cirrhosis, or other perceived overload risks.

From an ED perspective, this bedside role can be understood through several recurring physiologic patterns rather than through fixed treatment categories. When FoCUS suggests preserved left ventricular systolic function, there is no clear right ventricular strain, and PLR-VTI supports responsiveness without increasing evidence of pulmonary or venous congestion, a cautious additional fluid bolus may be reasonable in selected patients. By contrast, when FoCUS suggests myocardial dysfunction, right ventricular strain, or progressively rising pulmonary or systemic venous congestion, vasopressors, closer reassessment, or a more restrictive strategy may warrant consideration before additional empiric fluid. In other patients, the overall picture may be more consistent with vasoplegia, in which persistent hypotension after an initial fluid step may be more compatible with earlier norepinephrine support than with repeated fluid loading (28, 46). These patterns are intended as bedside interpretive frames rather than prescriptive treatment rules.

These profiles should not be understood as fixed categories. Sepsis physiology evolves over time, sometimes over the course of a single ED encounter, which is why any initial fluid or vasopressor choice should be revisited with serial ultrasound reassessment rather than treated as a one-time decision. Framed in this way, POCUS does not determine fluid strategy on its own; instead, it helps align bedside decisions more closely with the patient's current physiologic state.

### 5.5 The ultrasound resuscitation loop: serial reassessment as a safety feature

The ED is a physiologically noisy environment. Tachycardia, spontaneous respiratory effort, mechanical ventilation, pain, and vasopressor exposure can all distort the interpretation of static measurements. In this setting, one of the most important strengths of POCUS is not simply that it can be performed at the bedside, but that it can be repeated with minimal delay as the hemodynamic picture evolves. This repeatability is especially valuable in sepsis, where the relevant physiologic profile may shift over minutes rather than hours. At the same time, serial reassessment should not be confused with proof of improved outcomes; it is best viewed as a safety-oriented approach for selected patients in whom a specific management decision is uncertain.

A practical resuscitation loop begins with a baseline assessment using FoCUS, LUS, and, when feasible, dynamic flow-based testing. A targeted intervention can then be made, whether that intervention is a cautious fluid bolus, passive leg raise followed by a fluid decision, vasopressor initiation or escalation, inotropic evaluation, or ventilatory adjustment. Reassessment should be tied to that intervention rather than performed at arbitrary intervals. After a fluid bolus or vasopressor adjustment, a repeat check after approximately 5–15 min can ask whether VTI or other stroke-volume surrogates improved, whether oxygenation or B-line burden worsened, and whether venous congestion signals are emerging when advanced Doppler is used. If the answer is favorable, the strategy may continue cautiously; if perfusion does not improve or congestion rises, the next step should be to stop, switch, or escalate rather than give another empiric bolus.

This iterative, physiology-based approach is more useful than a one-time scan used to justify a fixed management plan (10, 12). Emerging physiologic work suggests that integrating cardiac, pulmonary, and selected venous information may help clinicians recognize when additional fluid is becoming less useful and more likely to contribute to positive fluid balance without meaningful benefit (30, 31, 37–39). However, the loop should still be understood mainly as a safety-oriented framework for serial bedside reassessment rather than as a prospectively validated workflow with proven outcome benefit in ED sepsis.

### 5.6 Implementation in the ED: training, reporting, governance, and common pitfalls

The suggested workflow outlined above requires implementation safeguards. Even well-conceived ultrasound frameworks are unlikely to improve care if protocols are excessively complex, poorly standardized, or inconsistently documented. For that reason, sepsis-oriented POCUS programs should remain brief, reproducible, and structured in a way that clearly separates source findings, shock phenotype, and fluid responsiveness or tolerance from one another (8–10, 12). This distinction reduces the risk that a single ultrasound impression will be overextended into conclusions it cannot reliably support. A minimum implementation framework is summarized in Table 5.

**Table 5:** Minimum implementation requirements for routine sepsis-oriented POCUS use in the emergency department.

Implementation domain	Minimum requirement for routine ED use
Framework structure	Time-boxed source-first scan plus FoCUS and LUS; PLR-LVOT VTI when Doppler skill is adequate; venous Doppler/VExUS only as optional advanced add-ons
Competency	Supervised scans with image review; emphasis on decision triggers, reliability limits, and escalation rules
Documentation	Structured note or worksheet that separates source findings, phenotype, and responsiveness/tolerance assessments; saved key clips when feasible
Quality assurance	Regular audit of image adequacy, diagnostic accuracy, escalation appropriateness, and selected workflow/process outcomes
Escalation and governance	Clear rules for CT, radiology-performed ultrasound, or formal echocardiography when uncertainty, poor image quality, or high-risk features persist

CT, computed tomography; ED, emergency department; FoCUS, focused cardiac ultrasound; LUS, lung ultrasound; LVOT, left ventricular outflow tract; PLR, passive leg raise; POCUS, point-of-care ultrasound; VExUS, venous excess ultrasound; VTI, velocity-time integral.

Training should prioritize acquisition of interpretable images, recognition of reliability limits, and the use of explicit decision triggers rather than attempting to reproduce comprehensive echocardiography at the bedside. For most ED programs, a sensible minimum dataset may include a trigger-based source scan, FoCUS, and LUS. PLR-LVOT VTI may be added when Doppler skill and image quality are adequate. Venous Doppler or the VExUS framework should be treated as optional advanced add-ons rather than routine requirements for all ED sepsis encounters. The main technique domains discussed in this review are illustrated in Figure 2.

**Figure 2:**
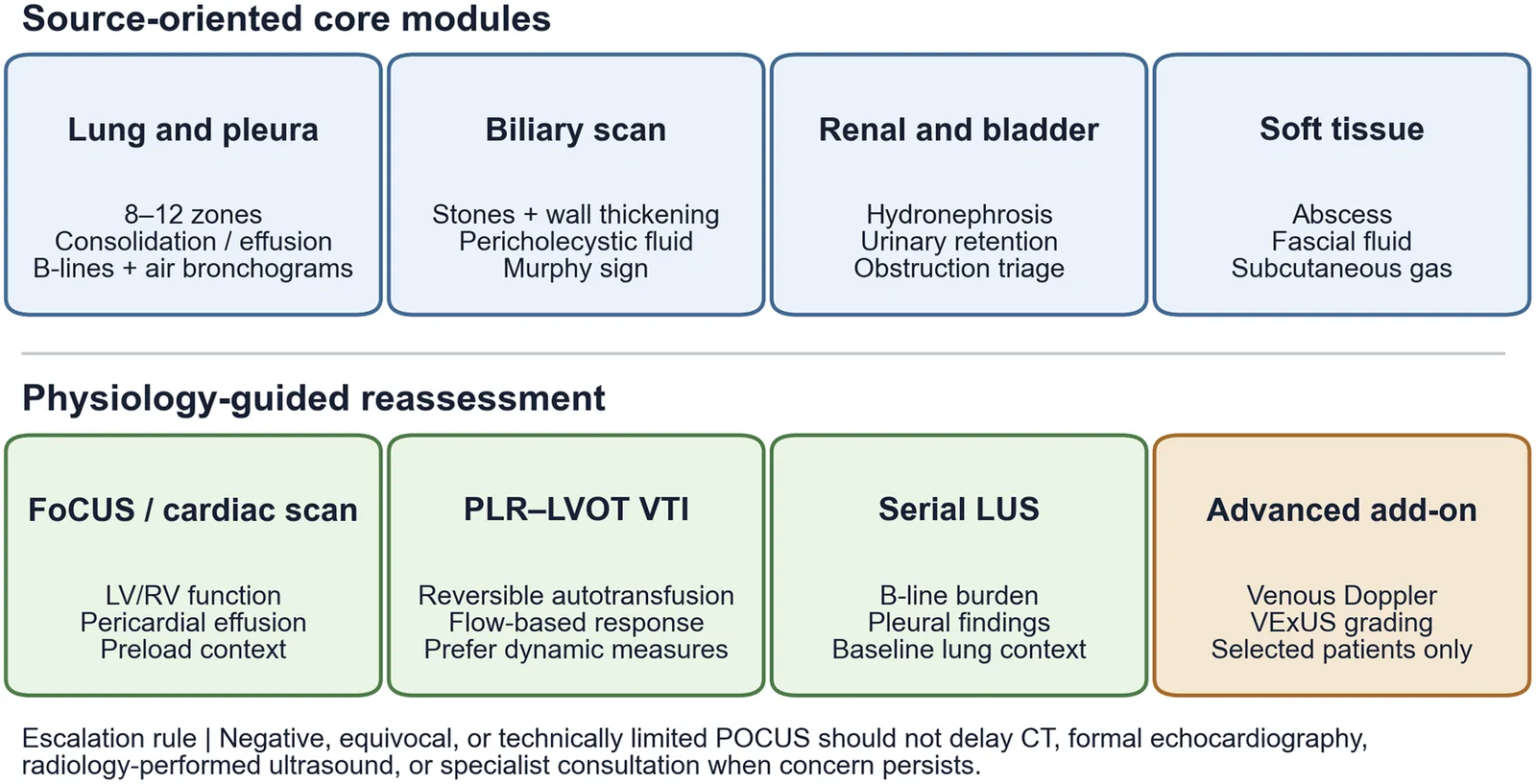
Core and advanced POCUS domains in the proposed ED sepsis framework. Source-oriented and physiology-guided core modules are trigger-based and decision-linked; venous Doppler/VExUS is retained as an optional advanced assessment for selected patients with adequate expertise and image quality. ED, emergency department; FoCUS, focused cardiac ultrasound; LUS, lung ultrasound; LV, left ventricle; LVOT, left ventricular outflow tract; PLR, passive leg raise; POCUS, point-of-care ultrasound; RV, right ventricle; VExUS, venous excess ultrasound; VTI, velocity-time integral.

## 6 Evidence limitations and risk of overinterpretation

The most important limitation of the current evidence base is that diagnostic capability, physiologic plausibility, and clinical utility are not equivalent. LUS may detect consolidation, soft-tissue ultrasound may detect abscess, FoCUS may identify ventricular dysfunction, and Doppler techniques may estimate fluid responsiveness or congestion. These capabilities can clarify bedside physiology and may trigger escalation, but they do not by themselves establish that a POCUS-guided ED sepsis pathway improves mortality, organ support, source-control timing, length of stay, or other patient-centered outcomes. Many of the available studies are diagnostic accuracy studies, focused reviews, physiologic investigations, ICU or perioperative studies, or observational analyses rather than ED sepsis-specific interventional trials. The evidence profiles for the principal POCUS applications in adult ED sepsis and septic shock are summarized in Table 6.

**Table 6:** Evidence profile for principal POCUS applications in adult ED sepsis and septic shock.

Application	Evidence profile	Main supporting studies	Current clinical role
Lung and pleural ultrasound	Moderate diagnostic evidence; adult pneumonia meta-analysis and diagnostic studies; direct outcome evidence remains limited.	Reviews and representative studies (8, 12, 15, 17–20)	Support a clinically concordant respiratory source, identify pleural complications, or reveal competing pulmonary physiology; do not replace CT/formal imaging when uncertainty persists.
Biliary, renal/bladder, and soft-tissue POCUS	Moderate rule-in diagnostic evidence for selected targets; heterogeneous indications and little evidence for patient-centered outcome benefit.	Systematic reviews and observational studies (21–25)	Trigger formal imaging, drainage planning, urologic/biliary decompression, surgical consultation, or source-control escalation in clinically selected patients.
FoCUS shock phenotyping	Supportive physiologic and diagnostic evidence; ED shock trials are not sepsis-specific and have not shown consistent short-term outcome improvement.	ED shock trials and focused reviews (29, 30, 32–34)	Identify major ventricular dysfunction, RV loading, pericardial disease, or a vasoplegic-dominant pattern as context for reassessment and escalation.
PLR with LVOT VTI or aortic flow surrogate	Moderate physiologic evidence for fluid responsiveness; heterogeneous modalities and limited direct ED sepsis outcome evidence.	Physiologic reviews and systematic synthesis (31, 34–36)	Estimate whether stroke volume is likely to rise; responsiveness alone is not an indication to administer fluid.
Serial LUS congestion assessment	Supportive physiologic and observational evidence; findings are nonspecific and treatment-outcome evidence is limited.	Consensus, physiologic, and review evidence (8, 19, 20, 31)	Track pulmonary interstitial burden and fluid tolerance alongside perfusion need, baseline lung disease, and ventilator effects.
Venous Doppler/VExUS	Emerging, predominantly observational and ICU-based evidence; ED sepsis data, interobserver reliability, and treatment validation remain limited.	Development, conceptual, and pilot cohort studies (37–39)	Optional advanced assessment in selected patients with adequate expertise and image quality; not a routine ED sepsis requirement.

Evidence categories are qualitative and reflect study design, consistency, ED specificity, and directness to the bedside question; they are not formal GRADE ratings. CT, computed tomography; ED, emergency department; FoCUS, focused cardiac ultrasound; GRADE, Grading of Recommendations Assessment, Development and Evaluation; ICU, intensive care unit; LUS, lung ultrasound; LVOT, left ventricular outflow tract; PLR, passive leg raise; POCUS, point-of-care ultrasound; RV, right ventricle/right ventricular; VExUS, venous excess ultrasound; VTI, velocity-time integral.

The evidence base is also limited by substantial heterogeneity in study populations, clinical settings, ultrasound targets, operator training, acquisition protocols, reference standards, and reported outcomes. Most supporting studies are observational, diagnostic, or physiologic, with relatively few randomized trials and limited ED sepsis-specific data. POCUS is inherently operator dependent, and performance may vary according to equipment capability, image quality, clinician experience, local training infrastructure, and institutional access to confirmatory imaging or specialist support. The absence of standardized multiorgan scanning and reporting protocols across studies further limits reproducibility and generalizability. The non-prespecified, iterative supplementary searches used to identify hemodynamic literature may also have introduced selection bias because complete search histories and aggregate yields were not retained.

For this reason, the framework presented here should be interpreted as a structured set of practice considerations, not as a clinical guideline or treatment protocol. Each ultrasound result should be linked to a narrow bedside question: whether a plausible source requires formal imaging or consultation, whether additional fluid is likely to increase flow, whether the patient appears fluid intolerant, or whether an alternative shock phenotype should be considered. When uncertainty persists, when image quality is poor, or when high-risk features are present, escalation to CT, radiology-performed ultrasound, formal echocardiography, and specialist involvement remains essential. Future trials are needed to determine whether predefined POCUS-triggered actions improve patient-centered outcomes in ED sepsis and septic shock.

## 7 Future directions

Several important gaps remain. The most pressing need is for ED-based trials that test whether sepsis-specific POCUS pathways can improve patient-centered outcomes rather than process measures or diagnostic confidence alone. Future studies should examine whether predefined ultrasound-triggered actions—for example, escalation to source-control imaging, withholding additional fluid in the setting of early congestion, or earlier vasopressor initiation after an initial fluid step—can improve outcomes when embedded within routine ED sepsis care. More reproducible and clinically meaningful congestion metrics are also needed in sepsis, particularly if pulmonary and venous ultrasound findings are to be incorporated more consistently into bedside fluid decisions.

Future research should also address how multiorgan POCUS can be integrated into everyday ED workflow without delaying antimicrobial therapy, vascular access, or other core elements of time-sensitive sepsis care. Beyond workflow alone, an additional priority will be to determine how ultrasound findings can be combined more reliably with perfusion markers, laboratory data, and emerging forms of electronic decision support. Prospective evaluation of serial ultrasound reassessment protocols with explicit stop/switch thresholds for fluid administration, vasopressor escalation, or escalation to source-control imaging would also be valuable. Progress in these areas will be essential if sepsis-focused POCUS is to evolve from a promising bedside framework into a reproducible and scalable component of emergency care.

## 8 Conclusions

Current evidence supports multiorgan point-of-care ultrasound primarily as an adjunctive bedside decision-support modality in adult ED sepsis and septic shock. Its greatest value lies in facilitating timely source localization, physiologic phenotyping, and individualized reassessment rather than replacing comprehensive imaging or established sepsis care pathways. POCUS should therefore complement, rather than delay, early antimicrobial therapy, source control, formal imaging, or specialist consultation. Evidence supporting patient-centered outcome improvement remains limited, especially for hemodynamic pathways and advanced congestion techniques.

Accordingly, the proposed two-arm approach should be understood as a pragmatic framework for selected bedside questions, not as a validated treatment protocol. Its safe use depends on appropriate patient selection, operator competency, adequate equipment and image quality, serial reassessment, local governance, and escalation when findings are negative, equivocal, or discordant with the clinical picture. Future prospective multicenter studies are needed to determine whether POCUS-guided diagnostic and resuscitation strategies translate into improved patient-centered outcomes.
