# Supplementary material for: Point-of-care ultrasound in emergency department sepsis and septic shock: a pragmatic framework for source-oriented assessment and physiologic reassessment
Source: Front Med (Lausanne). 2026 Sep 22;13:1919161. doi: 10.3389/fmed.2026.1919161 (PMC13639736; doi:10.3389/fmed.2026.1919161)
Supplement: Supplementary file 1 [file Table1.docx]

Supplementary Table S1. Database search strategies

Literature coverage: database inception to 6 May 2026. Final Search A date: 6 May 2026. No language restrictions were applied.

Search strategy note. Search A was the reproducible core strategy and required three concept blocks: sepsis/septic shock, ultrasound/POCUS, and source-oriented terms. The database-specific Search A strategies were run on 6 May 2026 and yielded 175 records in PubMed, 3,305 in Embase, 3,106 in Scopus, and 469 in Web of Science. Non-prespecified, iterative supplementary searches were also conducted across these databases to identify landmark hemodynamic literature. They combined sepsis/septic shock with topic-specific terms for focused cardiac ultrasound or echocardiography, passive leg raising/LVOT VTI/fluid responsiveness, lung ultrasound/B-lines/fluid tolerance, and venous Doppler/VExUS/venous congestion, together with backward and forward citation chaining. Because these exploratory searches were not protocolized, complete search histories and aggregate yields were not prespecified or retained and are not presented as fully reproducible systematic searches.

| **Database / platform** | **Coverage and filters** | **Final operational Search A Boolean string** | **Final Search A (6 May 2026)** |
| --- | --- | --- | --- |
| PubMed | Literature coverage: inception to 6 May 2026. No language restrictions were applied. | (("Sepsis"[Mesh] OR sepsis[tiab] OR "septic shock"[tiab]) AND ("Ultrasonography"[Mesh] OR ultrasound[tiab] OR ultrasonograph*[tiab] OR "point-of-care ultrasound"[tiab] OR "point of care ultrasound"[tiab] OR POCUS[tiab] OR "bedside ultrasound"[tiab] OR "lung ultrasound"[tiab]) AND ("source control"[tiab] OR pneumonia[tiab] OR pleural[tiab] OR empyema[tiab] OR biliary[tiab] OR cholecystitis[tiab] OR cholangitis[tiab] OR hydronephrosis[tiab] OR pyelonephritis[tiab] OR "urinary retention"[tiab] OR "urinary obstruction"[tiab] OR abscess[tiab] OR "soft tissue infection"[tiab] OR "necrotizing fasciitis"[tiab])) | Search completed on 6 May 2026; 175 records retrieved. |
| Embase | Literature coverage: inception to 6 May 2026. No language restrictions were applied. | (('sepsis'/exp OR sepsis:ti,ab,kw OR 'septic shock'/exp OR 'septic shock':ti,ab,kw) AND ('ultrasonography'/exp OR ultrasound:ti,ab,kw OR ultrasonograph*:ti,ab,kw OR 'point of care ultrasound':ti,ab,kw OR pocus:ti,ab,kw OR 'bedside ultrasound':ti,ab,kw OR 'lung ultrasound':ti,ab,kw) AND ('source control':ti,ab,kw OR pneumonia:ti,ab,kw OR pleural:ti,ab,kw OR empyema:ti,ab,kw OR biliary:ti,ab,kw OR cholecystitis:ti,ab,kw OR cholangitis:ti,ab,kw OR hydronephrosis:ti,ab,kw OR pyelonephritis:ti,ab,kw OR 'urinary retention':ti,ab,kw OR 'urinary obstruction':ti,ab,kw OR abscess:ti,ab,kw OR 'soft tissue infection':ti,ab,kw OR 'necrotizing fasciitis':ti,ab,kw)) | Search completed on 6 May 2026; 3,305 records retrieved. |
| Scopus | Literature coverage: inception to 6 May 2026. No language restrictions were applied. | TITLE-ABS-KEY((sepsis OR "septic shock") AND (ultrasound OR ultrasonograph* OR "point-of-care ultrasound" OR "point of care ultrasound" OR POCUS OR "bedside ultrasound" OR "lung ultrasound") AND ("source control" OR pneumonia OR pleural OR empyema OR biliary OR cholecystitis OR cholangitis OR hydronephrosis OR pyelonephritis OR "urinary retention" OR "urinary obstruction" OR abscess OR "soft tissue infection" OR "necrotizing fasciitis")) | Search completed on 6 May 2026; 3,106 records retrieved. |
| Web of Science | Literature coverage: inception to 6 May 2026. No language restrictions were applied. | TS=((sepsis OR "septic shock") AND (ultrasound OR ultrasonograph* OR "point-of-care ultrasound" OR "point of care ultrasound" OR POCUS OR "bedside ultrasound" OR "lung ultrasound") AND ("source control" OR pneumonia OR pleural OR empyema OR biliary OR cholecystitis OR cholangitis OR hydronephrosis OR pyelonephritis OR "urinary retention" OR "urinary obstruction" OR abscess OR "soft tissue infection" OR "necrotizing fasciitis")) | Search completed on 6 May 2026; 469 records retrieved. |

Abbreviations and field codes: ab, abstract; exp, exploded subject heading; MeSH, Medical Subject Headings; kw, keyword; POCUS, point-of-care ultrasound; ti, title; tiab, title/abstract; TITLE-ABS-KEY, title, abstract, and keywords; TS, topic.
